# Sex, power and drugs: the murky world of malaria parasite mitochondria

**DOI:** 10.1186/1475-2875-13-S1-P11

**Published:** 2014-09-22

**Authors:** Giancarlo Biagini

**Affiliations:** 1 Liverpool School of Tropical Medicine, Liverpool, L3 5QA, UK

## 

It is hypothesised that intraerythrocytic malaria parasite metabolism is not just fulfilling the need for ATP generation, but is highly evolved to support rapid proliferation, similar to what is seen in other rapidly proliferating cells such as cancer cells. Evidence is presented that deregulated glycolytic activity coupled with impaired mitochondrial metabolism is a metabolic strategy to generate glycolytic intermediates essential for rapid biomass generation for schizogony.

The role of the parasite mitochondrion during key stages of the parasite life cycle makes it an attractive target for the development of novel prophylaxis, treatment and transmission blocking drugs. Using a targeted pharmacometabolomic approach, additional mitochondrial targets with therapeutic potential are identified and the potential of the development of inhibitors against these novel targets is discussed in the context of recent experiences with bc_1_ and dihydroorotate dehydrogenase-targeting drug development programmes and within the context of current target product profiles for the malaria elimination agenda.

